# Telbivudine versus entecavir in patients with undetectable hepatitis B virus DNA: a randomized trial

**DOI:** 10.1186/s12876-017-0572-2

**Published:** 2017-01-19

**Authors:** Jihyun An, Young-Suk Lim, Gi-Ae Kim, Seong-bong Han, Wonhee Jeong, Danbi Lee, Ju Hyun Shim, Han Chu Lee, Yung Sang Lee

**Affiliations:** 1 Department of Gastroenterology, Liver Center, Asan Medical Center, University of Ulsan College of Medicine, 88, Olympic-ro 43-gil, Songpa-gu, Seoul, 05505 Korea; 2 Health Screening and Promotion Center, Asan Medical Center, Seoul, Republic of Korea; 3 Department of Applied Statistics, Gachon University, Seongnam-si, Gyeonggi-do Republic of Korea

**Keywords:** Hepatitis B surface antigen, HBsAg, Resistance, Virologic breakthrough, Virologic response

## Abstract

**Background:**

Telbivudine has been suggested to induce hepatitis B surface antigen (HBsAg) decline to the similar degree as pegylated interferon. We aimed to investigate whether telbivudine could further decrease HBsAg titer in patients who maintain undetectable serum hepatitis B virus (HBV) DNA after initial entecavir treatment.

**Methods:**

In this open-label trial, patients who had serum HBsAg and HBV DNA levels ≥1,000 IU/mL and <60 IU/mL, respectively, following entecavir (0.5 mg/day) treatment for HBeAg-positive chronic hepatitis B were randomized to either switch treatment to telbivudine (600 mg/day, *n* = 47) or continue entecavir (*n* = 50) for 48 weeks.

**Results:**

The baseline characteristics were comparable between groups including HBsAg levels (median, 3.41 log_10_ IU/mL). All patients had undetectable HBV DNA and normal alanine aminotransferase level. At week 48, the mean change in serum HBsAg levels was not significantly different between the telbivudine and entecavir groups (−0.03 log_10_ IU/mL *vs.* −0.05 log_10_ IU/mL; *P* = 0.57). No patient experienced HBsAg seroclearance or HBsAg decline >0.5 log_10_ IU/mL. Eleven patients (23.4%) in the telbivudine group, but none in the entecavir group, experienced virologic breakthrough (*P* < 0.001). Seven patients (14.9%) exhibited genotypic resistance mutations (M204I +/− L180M) during the virologic breakthrough.

**Conclusion:**

Sequential therapy with entecavir followed by telbivudine resulted in a high rate of virologic breakthrough and drug-resistance without any beneficial effect on HBsAg decline. These results do not support the use of low genetic barrier drugs as a switch treatment strategy in patients who achieve virologic response with high genetic barrier drugs.

**Trial registration:**

NCT01595685 (date of trial registration: May 8, 2012)

**Electronic supplementary material:**

The online version of this article (doi:10.1186/s12876-017-0572-2) contains supplementary material, which is available to authorized users.

## Background

Approximately 400 million people worldwide are chronically infected with hepatitis B virus (HBV). These patients have a substantially increased risk of cirrhosis and hepatocellular carcinoma (HCC), which are responsible for approximately 1 million deaths worldwide annually [1, 2]. The availability of potent nucleos(t)ide analogs (NUC) such as entecavir and tenofovir disoproxil fumarate (TDF) has made the suppression of serum HBV DNA to levels undetectable by polymerase chain reaction (PCR) assays achievable in most patients, with a minimal risk of drug-resistance [3, 4]. However, the eradication of HBV, which is best indicated by serum hepatitis B surface antigen (HBsAg) seroclearance, is rarely achievable with long-term NUC therapy [5–9]. The discontinuation of treatment before HBsAg seroclearance is associated with high rate of hepatitis relapse and disease progression [10, 11]. Therefore, treatments that can induce a rapid decline in HBsAg levels would have a clear advantage in reducing the treatment duration required to achieve HBsAg seroclearance.

Treatment with pegylated-interferon (PEG-IFN) has been reported to be associated with a greater HBsAg decline than NUC-treatment in patients with chronic HBV infection (CHB), regardless of hepatitis B envelope antigen (HBeAg) positivity [12, 13]. Interestingly, recent preliminary study demonstrated that telbivudine, a nucleoside analog, was associated with rapid HBsAg decline that was comparable to that induced by PEG-IFN in patients with HBeAg-positive CHB [14]. Although there has been no head-to-head trial comparing the induction of HBsAg decline by different NUCs, previous studies have repeatedly suggested that the decline in HBsAg may be greater during telbivudine treatment than it is with lamivudine or entecavir [12, 15–17]. Although telbivudine is associated with a relatively high rate of resistance, the risk could be reduced by profound early viral suppression to undetectable levels [18, 19].

In this randomised trial, we aimed to determine whether telbivudine induces a decline in HBsAg levels to a different degree compared with entecavir in patients with HBeAg-positive CHB, who have achieved undetectable serum HBV DNA levels by previous entecavir treatment.

## Methods

### Study design

**Fig. 1:**
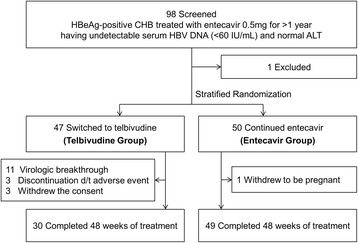
Patient flow diagram

This study was a randomized open-label trial (ClinicalTrials.gov ID NCT01595685; TERESA study) conducted in patients who had achieved a virologic response (serum HBV DNA <15 IU/mL) by preceding entecavir (0.5 mg once daily) treatment (Fig. 1). The patients were randomized (in a 1:1 ratio using a centralized procedure and an interactive web response system) to groups that either changed the treatment to telbivudine 600 mg once daily (Telbivudine group) or continued the entecavir treatment (Entecavir group) for 48 weeks.

The treatment assignments were generated by using a permuted block size of four after stratification based on serum HBsAg levels (1,000 − 5,000 *vs.* ≥5,000 IU/mL) and the duration of the preceding entecavir treatment (1–2 *vs.* ≥2 years). There was no interruption in entecavir therapy before randomization. This study was approved by the Institutional Review Board of the Asan Medical Center, and written informed consent was obtained from all study participants.

### Study subjects

The patients were enrolled between July 2012 and March 2013 at Asan Medical Center, an academic tertiary referral hospital in Korea. Patients were considered eligible for enrollment if they were positive for the HBeAg at the initiation of preceding entecavir treatment, received entecavir for more than 1 year, had undetectable serum HBV DNA levels (<60 IU/mL) on at least two occasions more than 3 months apart, and serum HBsAg levels >1,000 IU/mL at screening. Patients were required to be between 18 and 80 years old and have serum creatinine levels <1.5 mg/dL.

Patients were excluded if they met any of the following criteria: history of interferon therapy; prior exposure to oral antiviral agents other than entecavir for more than 1 week; evidence of decompensated liver disease; any malignant neoplasm; suspicion of HCC; received organ transplantation; concomitant use of immunosuppressive agent; or co-infection with hepatitis C, hepatitis D, or human immunodeficiency virus.

### Efficacy and safety assessments

The primary efficacy endpoint of this study was defined as a change in serum HBsAg levels from baseline to the end of week 48. The secondary endpoints were the proportions of patients with HBsAg loss/seroconversion, HBsAg decline ≥0.5 log_10_ IU/mL, HBeAg loss/seroconversion in those who were HBeAg-positive at randomization, and the incidence of virologic breakthrough (increases in HBV DNA levels ≥1 log_10_ IU/mL from nadir in two consecutive tests). The probability of developing genotypic resistance was assessed in all patients who experienced a virologic breakthrough or had viremia (i.e., HBV DNA >60 IU/mL) by the last time point of treatment and week 48.

Routine liver biochemistry, hepatitis B serology, and serum HBV DNA measurements were assessed at week 12, 24, and 48 after randomization. During each visit, patients were evaluated for adherence to study drugs by counting the number of pills and empty blister packets returned. The adverse events (clinical and laboratory) were assessed throughout the 48 weeks.

### Serum assays

The serum HBsAg levels were quantified by using the Architect assay (Abbott Laboratories, Chicago, IL, USA), which has a lower limit of detection (LLOD) of 0.05 IU/mL. Serum HBV DNA levels were measured using a real-time PCR assay (linear dynamic detection range, 15 IU/mL to 1 × 10^9^ IU/mL; Abbott Laboratories). Serological markers including anti-HBs, HBeAg, and anti-HBe were determined by using enzyme immunoassays (Abbott Laboratories) while resistance mutations were determined by direct sequencing of the reverse transcriptase domain (pol/RT) of the HBV polymerase gene. The HBV genotype was not determined because more than 98% of Korean patients with CHB have the HBV genotype C2 [20].

### Statistical analysis

The primary dataset for the efficacy and safety analyses was defined as all randomized patients. All the analyses were performed according to the intention-to-treat principle. Patients who discontinued the study prior to week 48 were considered failures for all endpoints from the time of discontinuation. The efficacy and safety analyses were performed by comparing the originally randomized Telbivudine and Entecavir groups.

The primary efficacy endpoint was the change in serum HBsAg levels at week 48. To observe a mean difference of 0.3 log_10_ IU/mL in the HBsAg decline between the Telbivudine and Entecavir groups with a two-sided 5% significance level and taking into account a dropout rate of up to 5%, an estimated 184 patients would have to be randomly assigned to each group to achieve 80% power. However, the study recruitment was discontinued after the inclusion of 97 patients because of slow accrual and identifying the significantly higher rate of virological breakthrough in the Telbivudine group at interim analysis.

The between-group comparisons of the continuous or categorical variables were conducted by using the *t*-test, Chi-square test, or Fisher’s exact test, as deemed appropriate. All the statistical analyses were performed by using the statistical package for the social sciences (SPSS, version 20, SPSS, Chicago, IL, USA) and R (version 3.0, http://cran.r-project.org/). A *P* < 0.05 was considered statistically significant.

## Results

### Baseline characteristics of patients

A total of 97 patients who had undetectable serum HBV DNA following entecavir treatment were randomly assigned to either the Telbivudine (*n* = 47) or Entecavir group (*n* = 50) as shown in Fig. 1.

Treatment groups were comparable in baseline demographic and laboratory characteristics (Table 1). The mean age was 47 years, and the population was predominantly male (69.1%). The median level of HBsAg was 3.41 log_10_ IU/mL. All patients were HBeAg-positive at the beginning of the preceding entecavir therapy; however, HBeAg positivity at randomization was 75.3%. All patients had an undetectable HBV DNA and normal alanine aminotransferase level. Thirty-four percent of the patients had cirrhosis. The median duration of prior entecavir treatment was 36 months.

**Table 1:** Baseline characteristics of the study patients

Characteristics	Total (*N* = 97)	Telbivudine (*n* = 47)	Entecavir (*n* = 50)
Age^*a*^, years	47 ± 10	48 ± 11	47 ± 10
Male, n (%)	67 (69.1%)	32 (68.1%)	35 (70.0%)
HBsAg^*b*^, log_10_ IU/mL	3.41 (3.15-3.69)	3.43 (3.17-3.84)	3.40 (3.10-3.67)
HBeAg positivity^*c*^, n (%)	73 (75.3%)	40 (85.1%)	33 (66.0%)
HBV DNA undetectable (<60 IU/mL), n (%)	97 (100%)	47 (100%)	50 (100%)
ALT^*b*^, IU/L	20 (16–30)	24 (16–31)	19 (14–27)
Bilirubin^*b*^, mg/dL	1.0 (0.8-1.2)	1.0 (0.7-1.3)	1.0 (0.8-1.2)
Albumin^*b*^, g/dL	4.4 (4.2-4.5)	4.3 (4.1-4.5)	4.4 (4.2-4.5)
INR^*b*^	0.98 (0.95-1.04)	0.98 (0.95-1.03)	0.98 (0.95-1.04)
Platelet^*b*^, ×1,000/mm^3^	167 (134–208)	170 (129–207)	166 (137–209)
Cirrhosis^*d*^, n (%)	33 (34.0%)	15 (31.9%)	18 (36.0%)
Creatinine^*b*^, mg/dL	0.9 (0.7-1.0)	0.9 (0.7-1.0)	0.9 (0.8-1.0)
Creatine kinase^*b*^, U/L	100 (77–129)	102 (81–156)	87 (72–116)
Duration of prior entecavir treatment^*b*^, months	36 (24–46)	33 (24–42)	39 (23–47)

^*a*^Mean ± standard deviation (SD)

^*b*^median (interquartile range)

^*c*^HBeAg positivity at randomization. All patients were HBeAg-positive at the beginning of preceding entecavir therapy

^*d*^Cirrhosis was diagnosed by using ultrasonography with identification of liver surface nodularity and splenomegaly

*HBsAg* hepatitis B surface antigen, *HBeAg* hepatitis B envelope antigen, *HBV* hepatitis B virus, *ALT* alanine aminotransferase, *INR* international normalized ratio

### Serologic responses

**Fig. 2:**
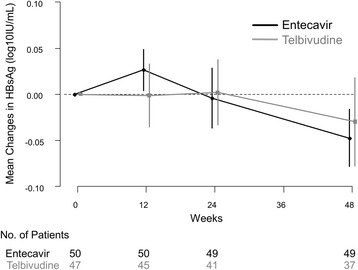
Changes in HBsAg levels from baseline

The mean change in serum HBsAg levels at week 48 of the treatment was not significantly different between the Telbivudine and Entecavir groups (−0.03 *vs.* -0.05 log_10_ IU/mL; *P* = 0.57; Table 2 and Fig. 2). No patient experienced HBsAg seroclearance or HBsAg decline >0.5 log_10_ IU/mL (Table 2). The proportion of patients who achieved HBsAg decline >0.1 log_10_ IU/mL was not significantly different between the Telbivudine and Entecavir groups (23.4% *vs.* 30.0%; *P* = 0.46). The proportion of HBeAg-positive patients who achieved HBeAg seroclearance was low without any significant difference between both groups at week 48 (5.0% *vs.* 15.2%; *P* = 0.14; Table 2). The serologic response at week 48 was not significantly different between the two groups by baseline HBeAg positivity, status of cirrhosis, and gender. An additional file showed these results in more detail [see Additional file 1].

**Table 2:** Serological, virological, and biochemical responses at week 48

Variables	Telbivudine (*n* = 47)	Entecavir (*n* = 50)	*P*-value
Serologic Responses			
Change in HBsAg level from baseline^*a,b*^, log_10_ IU/mL	−0.03 ± 0.14	−0.05 ± 0.11	0.57
HBsAg level^*a, c*^, log_10_ IU/mL	3.37 (3.22 - 3.63)	3.39 (3.10 - 3.67)	0.65
HBsAg seroclearance, n (%)	0 (0%)	0 (0%)	NA
HBsAg level decline from baseline >0.5 log_10_ IU/mL, n (%)	0 (0%)	0 (0%)	NA
HBsAg level decline from baseline >0.1 log_10_ IU/mL, n (%)	11 (23.4%)	15 (30.0%)	0.46
HBeAg seroclearance^*d*^, n (%)	2/40 (5.0%)	5/33 (15.2%)	0.14
HBeAg seroconversion^*d*^, n (%)	0/40 (0%)	2/33 (6.1%)	0.11
Virologic Responses			
Virologic breakthrough, n (%)	11 (23.4%)	0 (0%)	<0.001
Genotypic resistance, n (%)	7 (14.9%)	0 (0%)	0.005
Virologic response at week 48, n (%)	30 (63.8%)	49 (98.0%)	<0.001

Missing values were considered as failure for categorical endpoints

^*a*^Among participants whose serum HBsAg and HBV DNA level at week 48 was available (*n* = 37 in the Telbivudine group, *n* = 49 in the Entecavir group)

^*b*^Mean ± standard deviation (SD)

^*c*^Median (interquartile range)

^*d*^Among HBeAg-positive patients at randomization (*n* = 73)

*HBsAg* hepatitis B surface antigen, *HBeAg* hepatitis B envelope antigen, *NA* not applicable

### Virologic responses

Over the 48-week treatment period, 11 patients who were all in the Telbivudine group experienced virologic breakthrough (23.4%; *P* < 0.001; Table 2). All those had good adherence to study medication (>95%). Of these patients, genotypic resistance mutations to Telbivudine (M204I +/− L180M) were detected in seven during the virologic breakthrough. The detailed characteristics of the 11 patients are shown in Additional file 1. All of the patients recovered virologic response in 12 weeks following the administration of TDF or entecavir rescue therapy.

**Fig. 3:**
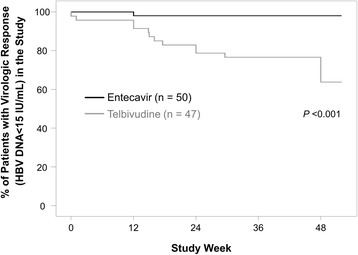
The proportion of patients maintaining virologic response (HBV DNA <60 IU/mL) in the study. Patients who discontinued the study prior to week 48 for any reason were considered failures in virologic response at the time of discontinuation

At week 48, the proportion of patients who maintained the virologic response in the study was significantly lower in the Telbivudine group than it was in the Entecavir group (63.8% vs 98.0%; *P* < 0.001, Table 2 and Fig. 3).

### Safety profiles

Three patients in the Telbivudine group discontinued the study because of headache, gastrointestinal trouble, and myopathy at week 1, 24, and 48, respectively. The symptoms improved after switching the drug to entecavir (Table 3). An elevation in serum creatine kinase (CK) levels >3 times of upper limit of normal (ULN, 250 IU/mL) was observed in three (6.4%) patients who all belonged to the Telbivudine group. A patient in the Telbivudine group experienced myopathy accompanied with elevated CK (920 IU/L) at week 48. The symptom improved and the serum CK level was normalized after the telbivudine was discontinued.

**Table 3:** Safety profiles of the study patients

Adverse event category	Telbivudine (*n* = 47)	Entecavir (*n* = 50)	*P*-value
Any adverse event	29 (61.7%)	29 (58.0%)	0.71
Serious adverse events^*a*^	2 (4.3%)	1 (2.0%)	0.52
Discontinuation due to adverse event^*b*^	3 (6.4%)	0	0.11
Dose reduction due to adverse event	0	0	-
Deaths	0	0	-
Serum CK >3 x ULN	3 (6.4%)	0	0.11
Myopathy	1 (2.1%)	0	0.30
HCC^*c*^, n (%)	1 (2.1%)	0	0.30
Serum creatinine ≥0.5 mg/dL above baseline	0	0	-
eGFR <50 mL · min^−1^ · 1.73 m^(2)-1^	0	0	-

^*a*^Telbivudine group: cholangitis with intra-hepatic duct stone, hepatocellular carcinoma; Entecavir group: scrub typhus. None was determined to be related to study drug administration

^*b*^By headache, gastrointestinal issues, and myopathy (*n* = 1 each). The symptoms improved after discontinuation of the treatment

^*c*^HCC was diagnosed at week 36

*CK* creatine kinase, *ULN* upper limit of normal, *HCC* hepatocellular carcinoma, *eGFR* estimated glomerular filtration rate

## Discussion

In this randomized trial, we found that in the patients with HBeAg-positive CHB, who achieved undetectable serum HBV DNA with the preceding entecavir treatment, switching the treatment to telbivudine for 48 weeks was not associated with a greater reduction in serum HBsAg levels. By contrast, telbivudine treatment was associated with a 23.4% virologic breakthrough and 14.9% genotypic resistance. None of the patients in the Entecavir group experienced virologic breakthrough or drug-resistance. Overall, the rate of maintaining virologic response was significantly lower in the Telbivudine group than that in the Entecavir group at week 48.

To date, few head-to-head randomized trials have investigated whether various NUCs induce a decline in HBsAg levels to different degrees. In HBeAg-positive patients, the rate of HBeAg seroconversion is only approximately 20–35% even after long-term treatment with a potent NUC such as entecavir or TDF [10, 21]. Furthermore, even after HBeAg-loss or -seroconversion induced by a potent NUC, the suppression of serum HBV DNA to undetectable levels is sustained only in approximately 23–37% at 24 weeks after treatment is discontinued. Therefore, HBsAg seroclearance is currently regarded as an optimal endpoint of treatment with NUC [22, 23]. In fact, our previous study demonstrated that HBsAg seroclearance achieved after NUC treatment persists in most cases and is associated with favorable clinical outcomes during long-term off-treatment follow-up [5]. However, HBsAg seroclearance is very rarely achievable, and almost life-long treatment is required in most patients. Based on HBsAg kinetics, it has been estimated that the predicted median time to HBsAg loss in patients treated with lamivudine or entecavir is more than 30–52 years [15, 24, 25]. A recent randomized trial showed that even the combination of the potent NUCs, entecavir and TDF, was not associated with greater decline in HBsAg levels compared with entecavir monotherapy through 96 weeks of treatment.

It has been suggested that the decline in HBsAg levels during lamivudine or entecavir therapy is slower and less pronounced than it is during interferon treatment, despite its higher suppression of HBV DNA [12, 15, 24]. Interestingly, experimental reports have suggested that telbivudine shares some common clinical mechanisms of action with interferon including dynamic changes in Th1/Th2 type cytokines [26]. In a trial for patients with HBeAg-positive CHB who received telbivudine treatment for up to 3 years and maintained undetectable serum HBV DNA level, up to 71 and 57% of the patients achieved HBeAg-loss and HBeAg seroconversion, respectively [16]. Another trial consisting of treatment-naïve patients with HBeAg-positive CHB revealed that the rate of patients with rapid HBsAg decline (≥0.5 log_10_ IU/mL) in the telbivudine monotherapy group (41%) was comparable to that in the PEG-IFN monotherapy group (31%) [14]. An observational study in Hong Kong including various NUC-treated patients with an initial immune active phase showed a significant reduction of HBsAg only in the telbivudine treatment group [17]. Although it is well known that telbivudine is associated with a higher rate of drug-resistance, previous studies identified the association of early profound viral suppression with a very low rate of drug resistance during long-term treatment. In patients who achieved HBV DNA levels undetectable in the quantitative PCR assay at week 24, the resistance risk at week 104 was only 4% [19]. However, the results of our current study contrast strikingly with our hypothesis. Switching the treatment of patients with virologic response induced by preceding entecavir treatment to telbivudine was associated with an unacceptably high rate of virologic breakthrough and drug-resistance without any beneficial effect on the HBsAg decline. This rate of virologic breakthrough (23.4%) during the 48-week telbivudine therapy in this study was comparable to that in a previous trial, which showed a 28.8% virologic breakthrough during a 2-year telbivudine treatment regimen in patients who were HBeAg-positive [19]. This observed rate is also similar to the rate of virologic rebound (24%) observed during lamivudine treatment in patients who had achieved undetectable serum HBV DNA following the preceding entecavir treatment [27].

The majority of our study patients did not exhibit HBeAg-seroclearance after the preceding >1-year entecavir therapy, which might have hindered the observation of a decline in the HBsAg levels. The HBsAg levels have been shown to decline rapidly during the first year of treatment [28]. Moreover, in HBeAg-positive patients, the decline in serum HBsAg is mainly confined to those who experience a clearance of HBeAg by either PEG-IFN or entecavir treatment [12]. However, because a high serum HBV DNA level is a strong predictor of the development of telbivudine-resistance, comparing telbivudine and entecavir in treatment-naïve patients could not be ethically justified.

This study has several limitations that are worth mentioning. First, the small sample size and short duration may have decreased the statistical power of the study to observe the differences in the decline of HBsAg levels between the Telbivudine and Entecavir groups. Nevertheless, the significantly higher rate of virologic breakthrough in the Telbivudine group did not justify the continuation of the study. Second, this was an open-label study and blinding was not performed. Although objective endpoints (serologic and virologic determinations) were used and drug adherence was ascertained, the lack of blinding might have influenced the response of the study patients or biased the investigators in reporting the adherence and adverse events. Lastly, the HBV genotype of our study patients was not determined. This was because one of the inclusion criteria of this study was an undetectable serum HBV DNA level at screening. However, since almost all Korean patients with CHB have the C HBV genotype [29], the application of the results of this study may be limited, and not extrapolatable to patients with other HBV genotypes.

## Conclusions

In conclusion, in patients who have achieved undetectable serum HBV DNA by entecavir treatment, switching the treatment to telbivudine for 48 weeks resulted in an unacceptably high rate of virologic breakthrough and drug-resistance without any beneficial effect on HBsAg decline. Prior viral suppression by entecavir did not confer any significant advantage to patients who switched to telbivudine. These results do not support the use of low genetic barrier drugs as a switch treatment strategy in patients who achieve virologic response by high genetic barrier drugs.

## Additional file

Additional file 1:
**Table S1.** Serological, virological, and biochemical responses at week 48 by baseline HBeAg-positivity. **Table S2.** Serological, virological, and biochemical responses at week 48 by status of liver cirrhosis. **Table S3.** Serological, virological, and biochemical responses at week 48 by gender. **Table S4.** Characteristics of the patients at virologic breakthrough. (DOCX 51 kb)

## Abbreviations

CHB: Chronic hepatitis B
CK: Creatine kinase
HBeAg: Hepatitis B envelope antigen
HBsAg: Hepatitis B surface antigen
HBV: Hepatitis B virus
HCC: Hepatocellular carcinoma
LLOD: Lower limit of detection
NUC: Nucleos(t)ide analog
PCR: Polymerase chain reaction
PEG-IFN: Pegylated interferon
pol/RT: Polymerase/reverse transcriptase domain
TDF: Tenofovir disoproxil fumarate
ULN: Upper limit of normal
