# Supplementary material for: Telbivudine versus entecavir in patients with undetectable hepatitis B virus DNA: a randomized trial
Source: BMC Gastroenterol. 2017 Jan 19;17:15. doi: 10.1186/s12876-017-0572-2 (PMC5248511; doi:10.1186/s12876-017-0572-2)
Supplement: Additional file 1: — Table S1. Serological, virological, and biochemical responses at week 48 by baseline HBeAg-positivity. Table S2. Serological, virological, and biochemical responses at week 48 by status of liver cirrhosis. Table S3. Serological, virological, and biochemical responses at week 48 by gender. Table S4. Characteristics of the patients at virologic breakthrough. (DOCX 51 kb) [file 12876_2017_572_MOESM1_ESM.docx]

**Additional File 1**

**Telbivudine versus Entecavir in Entecavir-Pretreated Patients with Undetectable Hepatitis B Virus DNA**

**: A Randomized Trial**

**Jihyun An, Young-Suk Lim, et al.**

**Supplementary Table 1. Serological, virological, and biochemical responses at week 48 by baseline** **HBeAg-positivity**

| **Variables** | **Patients with HBeAg-positivity** | | | **Patients with HBeAg-negativity** | | | |
| --- | --- | --- | --- | --- | --- | --- | --- |
|  | **Telbivudine**  **(n=40)** | **Entecavir**  **(n=33)** | ***P*-value** | **Telbivudine**  **(n=7)** | | **Entecavir**  **(n=17)** | ***P*-value** |
| ***Serologic Responses*** | | | | | | | |
| Change in HBsAg level from baseline*^a,b^*, log_10_IU/mL | -0.02 ± 0.15 | -0.04 ± 0.10 | 0.42 | -0.09 ± 0.13 | | -0.06 ± 0.13 | 0.41 |
| HBsAg level*^a,c^*, log_10_IU/mL | 3.44 (3.28-3.67) | 3.39 (3.14-3.70) | 0.47 | 3.05 (3.02-3.34) | | 3.19 (2.75-3.66) | 0.62 |
| HBsAg seroclearance, n (%) | 0 (0%) | 0 (0%) | NA | 0 (0%) | | 0 (0%) | NA |
| HBsAg level decrease from baseline >0.5 log_10_ IU/mL, n (%) | 0 (0%) | 0 (0%) | NA | 0 (0%) | | 0 (0%) | NA |
| HBsAg level decrease from baseline >0.1 log_10_ IU/mL, n (%) | 7 (17.5%) | 9 (27.3%) | 0.32 | 4 (57.1%) | | 6 (35.3%) | 0.39 |
| HBeAg seroclearance*^d^*, n (%) | 2 (5.0%) | 5 (15.2%) | 0.14 | - | | - | - |
| HBeAg seroconversion*^d^*, n (%) | 0 (0%) | 2 (6.1%) | 0.11 | - | | - | - |
| ***Virologic Responses*** | | | | | | | |
| Virologic breakthrough, n (%) | 11 (27.5%) | 0 (0%) | 0.12 | 0 (0%) | 0 (0%) | | - |
| Genotypic resistance, n (%) | 7 (17.5%) | 0 (0%) | 0.003 | 0 (0%) | 0 (0%) | | - |
| Virologic response at week 48, n (%) | 23 (57.5%) | 33 (100.0%) | <0.001 | 7 (100.0%) | 16 (94.1%) | | >0.99 |

Missing values were considered as failure for categorical endpoints.

*^a^*Among participants whose serum HBsAg and HBV DNA level at week 48 was available (n=37 in the Telbivudine group, n=49 in the Entecavir group)

*^b^*Mean ± standard deviation (SD)

*^c^*median (interquartile range)

*^d^*among HBeAg-positive patients at baseline (n=73)

HBsAg, hepatitis B surface antigen; HBeAg, hepatitis B envelope antigen; NA, not applicable.

**Supplementary Table 2. Serological, virological, and biochemical responses at week 48 by status of liver cirrhosis**

| **Variables** | **Telbivudine** | **Entecavir** | ***P*-value** |
| --- | --- | --- | --- |
| **Patients with Liver Cirrhosis** | **n=15** | **n=18** |  |
| **Serologic Responses** |  |  |  |
| Change in HBsAg level from baseline*^a,b^*, log_10_IU/mL | -0.001 ± 0.19 | -0.03 ± 0.99 | 0.56 |
| HBsAg level*^a, c^*, log_10_IU/mL | 3.29 (2.90 - 3.44) | 3.16 (2.72 - 3.43) | 0.27 |
| HBsAg seroclearance, n (%) | 0 (0%) | 0 (0%) | NA |
| HBsAg level decline from baseline >0.5 log_10_ IU/mL, n (%) | 0 (0%) | 0 (0%) | NA |
| HBsAg level decline from baseline >0.1 log_10_ IU/mL, n (%) | 3 (20.0%) | 4 (22.2%) | 0.88 |
| HBeAg seroclearance*^d^*, n (%) | 0/14 (0%) | 4/12 (33.3%) | 0.03 |
| HBeAg seroconversion*^d^*, n (%) | 0/14 (0%) | 2/12 (16.7%) | 0.20 |
| **Virologic Responses** |  |  |  |
| Virologic breakthrough, n (%) | 5 (33.3%) | 0 (0%) | 0.01 |
| Genotypic resistance, n (%) | 4 (26.7%) | 0 (0%) | 0.03 |
| Virologic response at week 48, n (%) | 7 (46.7%) | 18 (100.0%) | <0.01 |
| **Patients without Liver Cirrhosis** | **n=32** | **n=32** |  |
| **Serologic Responses** |  |  |  |
| Change in HBsAg level from baseline*^a,b^*, log_10_IU/mL | -0.04 ± 1.23 | -0.06 ± 0.11 | 0.65 |
| HBsAg level*^a, c^*, log_10_IU/mL | 3.41 (3.26 - 3.67) | 3.46 (2.72 - 3.43) | 0.91 |
| HBsAg seroclearance, n (%) | 0 (0%) | 0 (0%) | NA |
| HBsAg level decline from baseline >0.5 log_10_ IU/mL, n (%) | 0 (0%) | 0 (0%) | NA |
| HBsAg level decline from baseline >0.1 log_10_ IU/mL, n (%) | 8 (25.0%) | 11 (34.4%) | 0.59 |
| HBeAg seroclearance*^d^*, n (%) | 2/26 (7.7%) | 1/21 (4.8%) | >0.99 |
| HBeAg seroconversion*^d^*, n (%) | 0/26 (0%) | 0/21 (0%) | NA |
| **Virologic Responses** |  |  |  |
| Virologic breakthrough, n (%) | 6 (18.8%) | 0 (0%) | 0.02 |
| Genotypic resistance, n (%) | 3 (9.4%) | 0 (0%) | 0.24 |
| Virologic response at week 48, n (%) | 23 (71.9%) | 31 (96.9%) | 0.01 |

Missing values were considered as failure for categorical endpoints.

*^a^*Among participants whose serum HBsAg and HBV DNA level at week 48 was available (n=37 in the Telbivudine group, n=49 in the Entecavir group)

*^b^*Mean ± standard deviation (SD)

*^c^*median (interquartile range)

*^d^*Among HBeAg-positive patients at randomization (n=73)

HBsAg, hepatitis B surface antigen; HBeAg, hepatitis B envelope antigen; NA, not applicable.

**Supplementary Table 3. Serological, virological, and biochemical responses at week 48 by gender**

| **Variables** | **Telbivudine** | **Entecavir** | ***P*-value** |
| --- | --- | --- | --- |
| **Male** | **n=32** | **n=35** |  |
| **Serologic Responses** |  |  |  |
| Change in HBsAg level from baseline*^a,b^*, log_10_IU/mL | -0.05 ± 0.15 | -0.06 ± 0.09 | 0.71 |
| HBsAg level*^a, c^*, log_10_IU/mL | 3.30 (3.21 - 3.61) | 3.28 (2.96 - 3.46) | 0.27 |
| HBsAg seroclearance, n (%) | 0 (0%) | 0 (0%) | NA |
| HBsAg level decline from baseline >0.5 log_10_ IU/mL, n (%) | 0 (0%) | 0 (0%) | NA |
| HBsAg level decline from baseline >0.1 log_10_ IU/mL, n (%) | 10 (31.3%) | 11 (31.4%) | >0.99 |
| HBeAg seroclearance*^d^*, n (%) | 2/27 (7.4%) | 5/24 (20.8%) | 0.23 |
| HBeAg seroconversion*^d^*, n (%) | 0/27 (0%) | 2/24 (8.3%) | 0.22 |
| **Virologic Responses** |  |  |  |
| Virologic breakthrough, n (%) | 6 (18.8%) | 0 (0%) | 0.01 |
| Genotypic resistance, n (%) | 4 (12.5%) | 0 (0%) | 0.05 |
| Virologic response at week 48, n (%) | 22 (68.8%) | 35 (100.0%) | <0.01 |
| **Female** | **n=15** | **n=15** |  |
| **Serologic Responses** |  |  |  |
| Change in HBsAg level from baseline*^a,b^*, log_10_IU/mL | 0.03 ± 0.10 | -0.01 ± 0.14 | 0.50 |
| HBsAg level*^a, c^*, log_10_IU/mL | 3.39 (3.25 - 3.78) | 3.64 (3.30 - 3.95) | 0.82 |
| HBsAg seroclearance, n (%) | 0 (0%) | 0 (0%) | NA |
| HBsAg level decline from baseline >0.5 log_10_ IU/mL, n (%) | 0 (0%) | 0 (0%) | NA |
| HBsAg level decline from baseline >0.1 log_10_ IU/mL, n (%) | 1 (6.7%) | 4 (26.7%) | 0.33 |
| HBeAg seroclearance*^d^*, n (%) | 0/13 (0%) | 0/9 (0%) | NA |
| HBeAg seroconversion*^d^*, n (%) | 0/13 (0%) | 0/9 (0%) | NA |
| **Virologic Responses** |  |  |  |
| Virologic breakthrough, n (%) | 5 (33.3%) | 0 (0%) | 0.04 |
| Genotypic resistance, n (%) | 3 (20.0%) | 0 (0%) | 0.22 |
| Virologic response at week 48, n (%) | 8 (53.3%) | 14 (93.3%) | 0.04 |

Missing values were considered as failure for categorical endpoints.

*^a^*Among participants whose serum HBsAg and HBV DNA level at week 48 was available (n=37 in the Telbivudine group, n=49 in the Entecavir group)

*^b^*Mean ± standard deviation (SD)

*^c^*median (interquartile range)

*^d^*Among HBeAg-positive patients at randomization (n=73)

HBsAg, hepatitis B surface antigen; HBeAg, hepatitis B envelope antigen; NA, not applicable.

**Supplementary table 4. Characteristics of the patients at virologic breakthrough**

| **No.** | **Group** | **Week** | **Age** | **Sex** | **HBeAg-positivity** | **Months of prior treatment** | **Months of VR prior to enrollment** | **HBV DNA levels**  **(log_10_ IU/mL)** | **HBV resistance mutations** | **ALT** | **Rescue therapy** | **Outcome** |
| --- | --- | --- | --- | --- | --- | --- | --- | --- | --- | --- | --- | --- |
| 1 | Telbivudine | 16 | 54 | M | + | 18 | 9 | 4.08 | M204I + L180M | 35 | TDF | VR |
| 2 | Telbivudine | 15 | 39 | F | + | 33 | 18 | 2.76 | M204I + L180M | 27 | TDF | VR |
| 3 | Telbivudine | 47 | 43 | F | + | 33 | 12 | 2.96 | M204I | 10 | TDF | VR |
| 4 | Telbivudine | 50 | 53 | M | + | 21 | 14 | 6.46 | M204I | 738 | TDF | VR |
| 5 | Telbivudine | 48 | 56 | F | + | 38 | 30 | 3.98 | M204I | 26 | TDF | VR |
| 6 | Telbivudine | 48 | 45 | M | + | 17 | 8 | 4.08 | M204I | 23 | TDF | VR |
| 7 | Telbivudine | 48 | 47 | M | + | 24 | 11 | 4.51 | M204I | 16 | TDF | VR |
| 8 | Telbivudine | 18 | 49 | M | + | 34 | 32 | 2.34 | None | 20 | TDF | VR |
| 9 | Telbivudine | 15 | 50 | F | + | 7 | 5 | 3.61 | None | 14 | Entecavir | VR |
| 10 | Telbivudine | 12 | 68 | F | + | 15 | 12 | 2.36 | None | 33 | Entecavir | VR |
| 11 | Telbivudine | 12 | 47 | M | + | 30 | 7 | 2.00 | None | 42 | Entecavir | VR |

HBeAg, hepatitis B envelope antigen; VR, virologic response; HBV, hepatitis B virus; ALT, alanine aminotransferase; TDF, tenofovir disoproxil fumarate.
